# EPMA quantification on the chemical composition of retained austenite in a Fe-Mn-Si-C-based multi-phase steel

**DOI:** 10.1186/s42649-022-00083-0

**Published:** 2022-12-20

**Authors:** Yoon-Uk Heo, Chang-Gon Jeong, Soo-Hyun Kim, Gun-Young Yoon, T. T. T. Trang, Youngyun Woo, Eun Yoo Yoon, Young-Seon Lee

**Affiliations:** 1 Graduate Institute of Ferrous and Energy Materials Technology, Pohang University of Science and Technology, Cheongam-Ro 77, Hyoja dong, Pohang, 37-673 Republic of Korea; 2 Korea Institute for Materials Science, 797 Changwon-daero, Seongsan-gu, Changwon, 51508 Republic of Korea

**Keywords:** EPMA quantification, Retained austenite, Multi-phase steel, XRD, Carbon content

## Abstract

An electron probe X-ray microanalyzer (EPMA) is an essential tool for studying chemical composition distribution in the microstructure. Quantifying chemical composition using standard specimens is commonly used to determine the composition of individual phases. However, the local difference in chemical composition in the standard specimens brings the deviation of the quantified composition from the actual one. This study introduces how to overcome the error of quantification in EPMA in the practical aspect. The obtained results are applied to evaluate the chemical composition of retained austenite in multi-phase steel. Film-type austenite shows higher carbon content than blocky-type one. The measured carbon contents of the retained austenite show good coherency with the calculated value from the X-ray diffraction.

## Introduction

An electron probe X-ray microanalyzer (EPMA) is a powerful equipment to characterize the distribution of the chemical composition in various materials (Rinaldi and Llovet 2015). EPMA determines the chemical composition accurately due to its high energy resolution (~ 10 eV) (Williams and Carter 2009). The elemental map confirms the relative intensity difference of individual elements in the various phases, including precipitate, inclusion, and matrix phases (Lee et al. 2022; Han et al. 2021). Although there was an effort to quantify the X-ray intensity without a standard specimen (Trincavelli et al. 2014), the obtained intensity is generally quantified by comparing it with the intensities of standard specimens where their chemical compositions have already been determined. The calibration curves that show the correlation between X-ray intensity and absolute composition for the individual elements should have to be obtained from the standard specimens under the same analysis condition (beam diameter, beam current, accelerating voltage, and detecting crystals) to the observation condition for the actual specimen. Since the chemical compositions of the standard specimens are varied to obtain the correct calibration curve, repeated EPMA analyses on the multiple numbers of standard specimens are necessary. The calibration curve is generally expressed by a linear equation below (Rinaldi and Llovet 2015);

1 $${\boldsymbol I}_{\boldsymbol i}\;\left(\textbf{counts}\right)={\boldsymbol k}_{\boldsymbol i}\times{\boldsymbol C}_{\boldsymbol i}\mathit{\left({\boldsymbol w\boldsymbol t.\%}\right)}+\textbf{B}$$

where ***I***_***i***_ is the X-ray counts of element i, ***C***_***i***_ is an absolute composition of element *i*, ***k***_***i***_ is the proportional constant, and **B** is a background intensity or an intensity without element *i* in the matrix. X-ray emission depends on atomic number (Z), the absorption of X-rays (A), and the fluorescence of X-rays within the specimen (F) (Williams and Carter 2009; Trincavelli et al. 2014; Ziebold and Ogilvie 2002). Therefore, the proportional constant ***k***_***i***_ is inversely proportional to the ZAF correction factor. ***k***_***i***_ and B values are varied with the corresponding element and measuring conditions (beam diameter, beam current, accelerating voltage, detecting crystals, and so on).

One of the typical difficulties in EPMA quantification is finding a proper standard specimen. Pure element is one of the candidates for the standard specimen. However, the absorption of X-rays and the efficiency of X-ray generation in multi-element materials are affected by the constituent elements. This brings the deviation from the exact composition when we study a diluted system using pure element standard. The proper standard specimens will be a form of a solid solution containing the compositions covering the range of interest. Since the chemical compositions of the standard specimens are diverse, the elemental distributions in the standard specimens are nonuniform. These compositional inhomogeneities in the standard specimens bring a significant deviation from the absolute composition of the target specimen because they make an uncorrected calibration curve. Since the chemical inhomogeneity of standard specimens is intrinsic and cannot avoid, the proper method to overcome it is necessary from a practical viewpoint.

The multi-phase steels are composed of diverse phases, including α-ferrite, α_b_-bainite, α′-martensite, pearlite, and retained austenite (γ) (Han et al. 2021; Kim et al. 2022a). Among them, γ controls the mechanical properties in the multi-phase steels. γ changes the work-hardening and ductility of the steel through the transformation-induced plasticity (TRIP) effect during mechanical deformation (Spencer et al. 2009). The TRIP phenomenon has a strong relationship with the mechanical stability of γ. The chemical compositions of γ that are specifically the contents of γ stabilizer (C, Mn, and so on) should be determined to evaluate the mechanical stability of γ (Heo et al. 2016). However, there are several difficulties to investigate γ in the multi-phase steels using transmission electron microscopy (TEM). Firstly, the volume fraction of γ is only a few % order. There is a limitation in finding the retained γ in a TEM specimen (Zhu et al. 2012). Rare distribution of γ is also an obstacle to fabricating TEM specimens using a focused ion beam. Secondly, statistical analysis is difficult in TEM analysis due to a limited observation of γ (Gutierrez-Urrutia et al. 2013).

In this study, we aim for the practical aspect of EPMA quantification of the chemical composition of retained γ in multi-phase steel. The accurate quantification method in EPMA analysis is suggested. Moreover, EPMA analysis’s obtained carbon (C) content was compared to the calculated result from the X-ray spectrometry (XRD).

## Material and methods

### Preparation of EPMA quantification

**Fig. 1:**
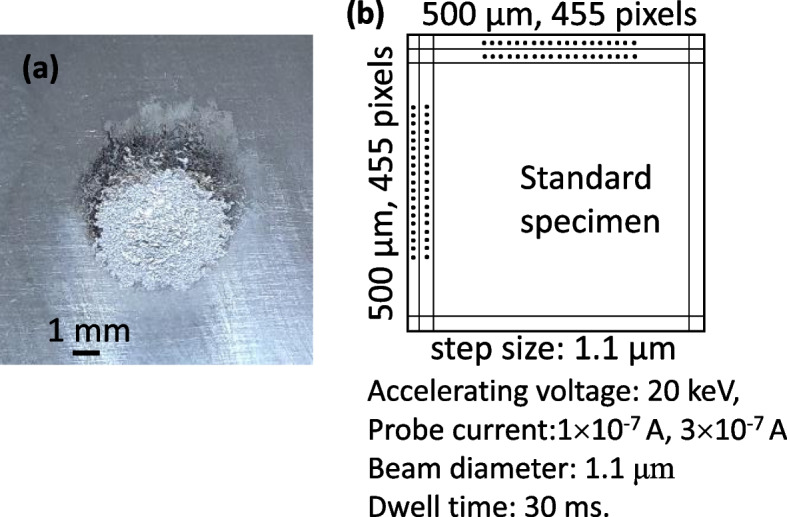
**a** Typical image of the specimen after testing the optical emission spectroscopy and **b** detailed acquisition condition of X-ray signal in EPMA mapping

The standard specimens were prepared, including a different amount of Mn, Si, and C. Except for 2.029Si alloy, the chemical compositions of all the standard specimens are verified by the National Institute of Standards and Technology. The details compositions are listed in Table 1. The standard specimens were mechanically polished with SiC papers. Then, micro-polishing was conducted using diamond suspensions holding 1, 3, and 9 μm particles. All the compositions of the standard specimens were also investigated by optical emission spectroscopy (OES) and confirmed finally (Table 1). EPMA (JXA-8530F, JEOL Ltd., Tokyo) mapping was conducted to obtain the accurate calibration curve. The condition for the EPMA mapping is given in Fig. 1.

**Table 1:** Chemical compositions of standard specimens (wt.%)

Standard specimens								
Element	SRM1761^a^	SRM1763^a^	SRM1764^a^	SRM1765^a^	SRM1767^a^	SRM C1151a	SRM C1153a	2.029Si
C	1.03	0.203	0.592	0.006	0.052	0.034	0.225	
Mn	0.678	1.58	1.21	0.144	0.022	2.37	0.544	
P	0.04	0.012	0.02	0.0052	0.0031	0.017	0.03	
S	0.035	0.023	0.012	0.0038	0.009	0.038	0.019	
Si	0.18	0.63	0.057		0.026	0.29	1	2.029
Others^b^	7.8674	7.5136	7.1326	0.6166	0.3101	64.8608	54.2867	
Fe	Balance	Balance	Balance	Balance	Balance	Balance	Balance	Balance

^a^The compositions of the specimens are verified by the National Institute of Standards and Technology

^b^Others are various elements, including Cu, Zn, Ni, V, Al, Pb, Sb, Ag, B, N, etc.

### Characterization of microstructure in a Fe-Mn-Si-C alloy

An alloy (Fe-1.5Mn-1.5Si-0.25C (wt.%)) was prepared by vacuum induction heating. First, the specimen was hot-rolled to a rod with a 20 mm diameter. Then, the intercritical annealing was performed at 800 °C for 20 min. The annealed specimen was cooled to 400 °C with 15 °C/s and then held at 400 °C for 30 min. α_b_ -bainite is formed during the isothermal holding. The retained γ is stabilized by the active C partitioning from α_b_ to γ at 400 °C. The microstructure of the heat-treated multi-phase steel was investigated using a field emission-scanning electron microscope (FE-SEM, JSM-7900F, JEOL Ltd., Tokyo) equipped with an electron backscatter diffraction (EBSD, Aztec, Oxford, Abingdon) detector. The chemical compositions of the retained γ were investigated using EPMA. XRD spectrum of the specimen was also obtained to measure the precise lattice parameter of γ (Cullity and Stock 2001).

## Results and discussion

### Effects of acquisition conditions on the calibration curves

**Fig. 2:**
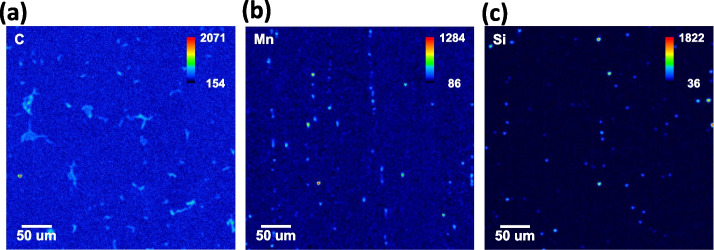
Compositional inhomogeneities in the standard specimens; **a** C Kα (SRM 1767), **b** Mn Kα (SRM 1761), and **c** Si Kα (SRMC 1151a) maps. The maps were obtained under 20 keV(Accelerating voltage) and 3 × 10^− 7^ A (Probe current)

As shown in Fig. 1a, OES analysis uses a large quantification area. Due to the large measuring areas, local compositional fluctuation is not sensitively affected by averaging the composition out. However, the compositional inhomogeneity in the standard specimen brings a critical error in constructing the calibration curve in EPMA. A few sampling points in a standard specimen give incorrect intensity values. As a result, there are significant deviations in X-ray intensities in a standard specimen, as shown in Fig. 2a to c. Since most standard specimens are carbon steels, the local difference in carbon intensity, which originated from different phases (cementite, carbide, retained γ or α′) in a standard specimen, is detected in Fig. 2a. This compositional inhomogeneity could be overcome by increasing the sampling area and averaging the obtained intensities. EPMA mapping was conducted using a relatively large area of a 500 μm × 500 μm area (Fig. 1b). All the intensities in a map are summed and then divided by the total number of pixels (455 pixels × 455 pixels).

**Fig. 3:**
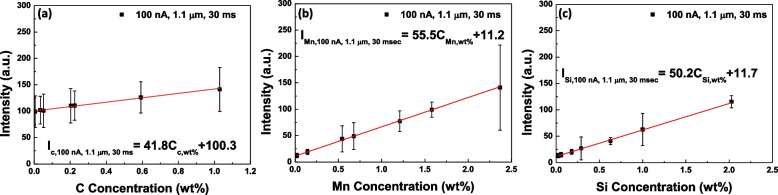
Calibration curves under the condition of 20 keV, 3 × 10^− 7^ A, 1.1 μm (beam diameter), and 30 ms (dwell time); **a** C Kα, **b** Mn Kα, and **c** Si Kα

Figure 3a to c show the constructed calibration curves from the various standard specimens in Table 1. The acquisition conditions of X-ray intensities in EPMA are 20 keV(accelerating voltage), 3 × 10^− 7^ A (probe current), 1.1 μm (beam diameter), and 30 ms (dwell time), respectively. Although the standard deviations of each specimen are significantly large, the average values show well-defined linearity. The obtained calibration curves for C, Mn, and Si are

2 $${\boldsymbol I}_{\boldsymbol C,\;\textbf{100}\;\boldsymbol{nA},\;\textbf{3}\;\textbf{0}\;\boldsymbol{ms}}=\textbf{41.8}\times{\boldsymbol C}_{\boldsymbol C}+\textbf{100}.\textbf{3},$$

3 $${\boldsymbol I}_{\boldsymbol{Mn},\;\textbf{100}\;\boldsymbol{nA},\;\textbf{3}\;\textbf{0}\;\boldsymbol{ms}}=\textbf{55.5}\times{\boldsymbol C}_{\boldsymbol{Mn}}+\textbf{11.2},$$

4 $$\text{and}\;{\boldsymbol I}_{\boldsymbol{Si},\;\textbf{100}\;\boldsymbol{nA},\;\textbf{3}\;\textbf{0}\;\boldsymbol{ms}}=\textbf{50.2}\times{\boldsymbol C}_{\boldsymbol{Si}}+\textbf{11.7}.$$

#### The effect of probe current change on the calibration curves

The various probe currents are used for characterizing coarse and fine microstructures in EPMA. All through the fine probe is beneficial in spatial resolution, a small primary electron results in less X-ray emission. The optimum condition of the probe current is determined by the trade-off between special resolution and the output intensity of the X-ray signal. The effect of probe current change on the calibration curves was studied using the same standard specimens.

**Fig. 4:**
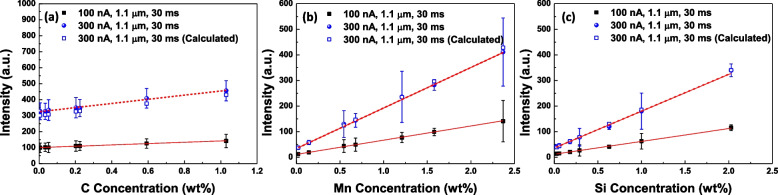
Calibration curves under the different probe currents; **a** C Kα, **b** Mn Kα, and **c** Si Kα. The calculated data were obtained from the intensity in 1 × 10^− 7^ A probe current by multiplying 3

Figure 4 shows the change in the calibration curves depending on the probe current. The obtained intensities at each standard specimen are almost three times higher when the probe current changes from 1 × 10^− 7^ A to 3 × 10^− 7^ A. The obtained calibration curves in 3 × 10^− 7^ A are

5 $${\boldsymbol I}_{\boldsymbol C,\;\textbf{300}\;\boldsymbol{nA},\;\textbf{3}\;\textbf{0}\;\boldsymbol{ms}}=\textbf{130.0}\times{\boldsymbol C}_{\boldsymbol C}+\textbf{324.2},$$

6 $${\boldsymbol I}_{\boldsymbol{Mn},\;\textbf{300}\;\boldsymbol{nA},\;\textbf{3}\;\textbf{0}\;\boldsymbol{ms}}=\textbf{158.3}\times{\boldsymbol C}_{\boldsymbol{Mn}}+\textbf{34.0},$$

7 $$\text{and}\;{\boldsymbol I}_{\boldsymbol{Si},\;\textbf{300}\;\boldsymbol{nA},\;\textbf{3}\;\textbf{0}\;\boldsymbol{ms}}=\textbf{143.7}\times{\boldsymbol C}_{\boldsymbol{Si}}+\textbf{36.2}.$$

The experimental data and calculated intensities obtained from the three times the intensities in the probe current of 1 × 10^− 7^ A condition were compared. As shown in Fig. 4a to c, the calculated intensities show coherency with the experimental data except for C in Fig. 4a. The probe current of 3 × 10^− 7^ A shows higher intensity than the calculated one. C is frequently stacked on the specimen surfaces during observation in electron microscopy (Kim et al. 2022b). Higher current density provides more chances to dehydrogenize the hydrocarbon on the specimen (Toth et al. 2009). It results in more C contamination during the acquisition of the X-ray signal. Therefore, higher intensity in the probe current of 3 × 10^− 7^ A is originated from the additional contribution of C-contamination. The increased ***k***_***i***_ in high probe current will be beneficial for the quantification by increasing the X-ray intensity gap between similar compositions.

#### The effect of dwell time on the calibration curves

**Fig. 5:**
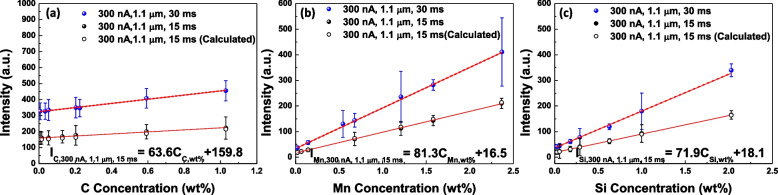
Calibration curves under the different dwell times; **a** C Kα, **b** Mn Kα, and **c** Si Kα. The calculated data were obtained from the intensity in 1 × 10^− 7^ A probe current and 30 ms by dividing 2

To reduce the C-contamination, a short dwell time for mapping is recommended. The effect of dwell time on the calibration curve was investigated in Fig. 5. The X-ray intensity reduces by about half when the dwell time decreases from 30 ms to 15 ms. The experimental data was compared to the calculated intensities obtained from half of the intensities in the 30 ms condition. Both intensity values show good coherency. Decreased or increased dwell time directly contributes to X-ray generation. Figures 4 and 5 show that a calibration curve can be converted into the calibration curves for the different acquisition conditions. The empirical formulation was drawn as below;

8 $${\boldsymbol{I}}_i^{\prime}\,\left({\textbf{p}}_{\textbf{2}},\, {\textbf{t}}_{\textbf{2}}\right)={\boldsymbol{I}}_{\boldsymbol{i}}\left({\textbf{p}}_{\textbf{1}},\, {\textbf{t}}_{\textbf{1}}\right)\times \left({\textbf{p}}_{\textbf{2}}/{\textbf{p}}_{\textbf{1}}\right)\times \left({\textbf{t}}_{\textbf{2}}/{\textbf{t}}_{\textbf{1}}\right)$$

where p_i_ is the probe current and t_i_ is the dwell time for the measurement.

### Quantification of the chemical composition of retained γ in a Fe-Mn-Si-C alloy

**Fig. 6:**
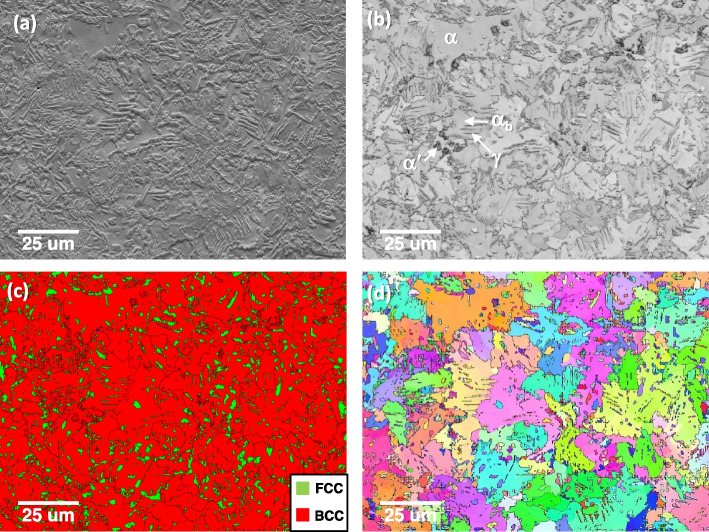
Microstructure of a Fe-1.5Mn-1.5Si-0.25C alloy; **a** secondary electron image, **b** Band contrast, **c** phase, and **d** inverse pole figure maps

**Fig. 7:**
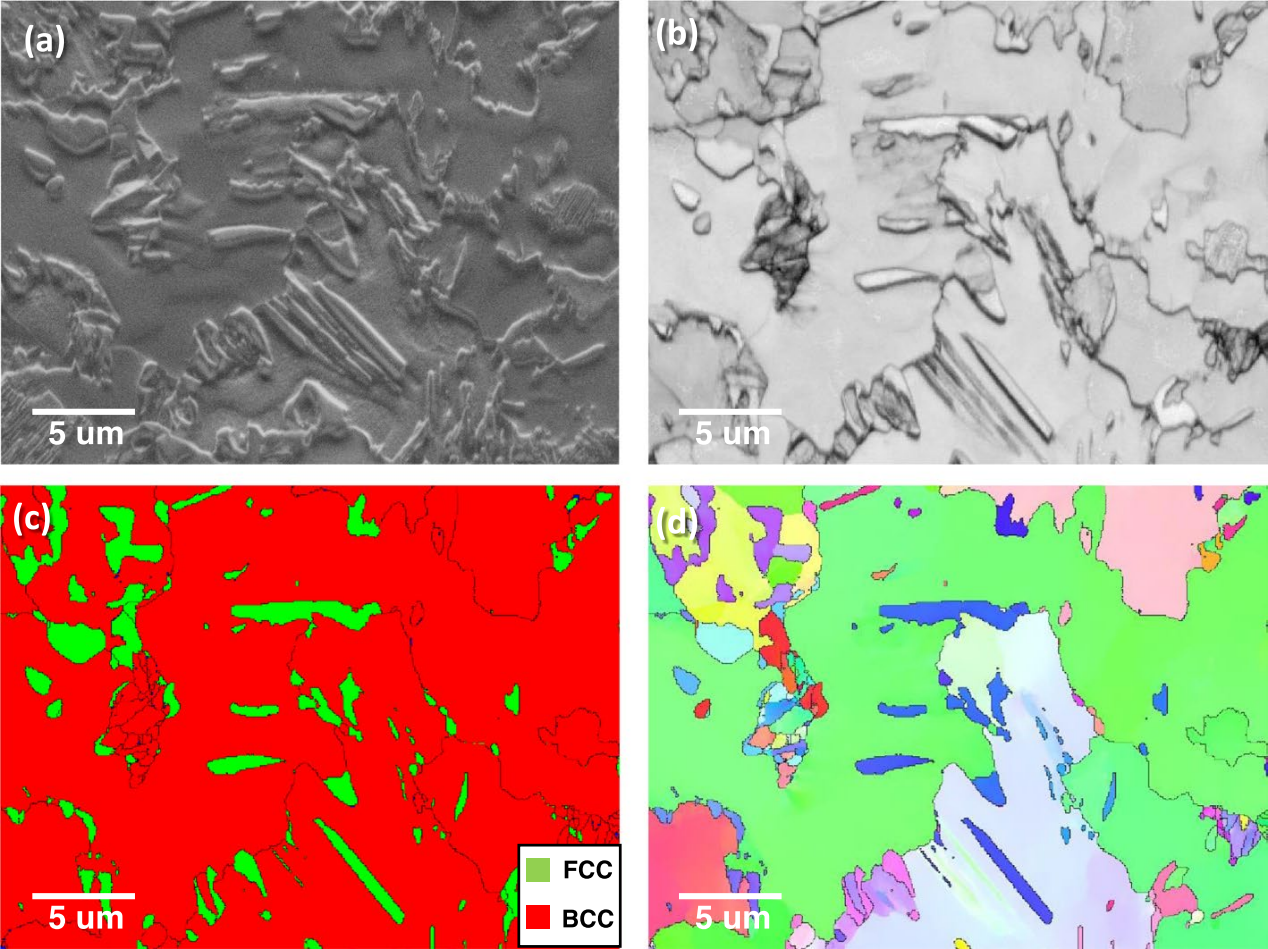
The morphology of retained γ (FCC) in high magnification; **a** secondary electron image, **b** band contrast, **c** phase, and **d** inverse pole figure maps

A Fe-1.5Mn-1.5Si-0.25C (wt.%) alloy shows a complex phase structure. Figure 6 shows a typical microstructure of the steel. A secondary electron (SE) image shows complex microstructures. The band contrast map reveals defect-lean α-ferrite(bright contrast) and defect-enriched α′-martensite or α_b_-bainite (dark contrast). The retained γ was also detected in the phase map in Fig. 6c. Since the alloy experienced intercritical annealing at 800 °C, the various orientations of α′/α_b_ in a α grain originated from the γ to α′/α_b_ transformation (Fig. 6d). The analysis was further performed to reveal the microstructure of γ. Figure 7a and b show the morphology of retained γ in high magnification. The retained γ shows protruding, clean, and elongated morphologies in SE and band contrast images (Fig. 7a to c).

**Fig. 8:**
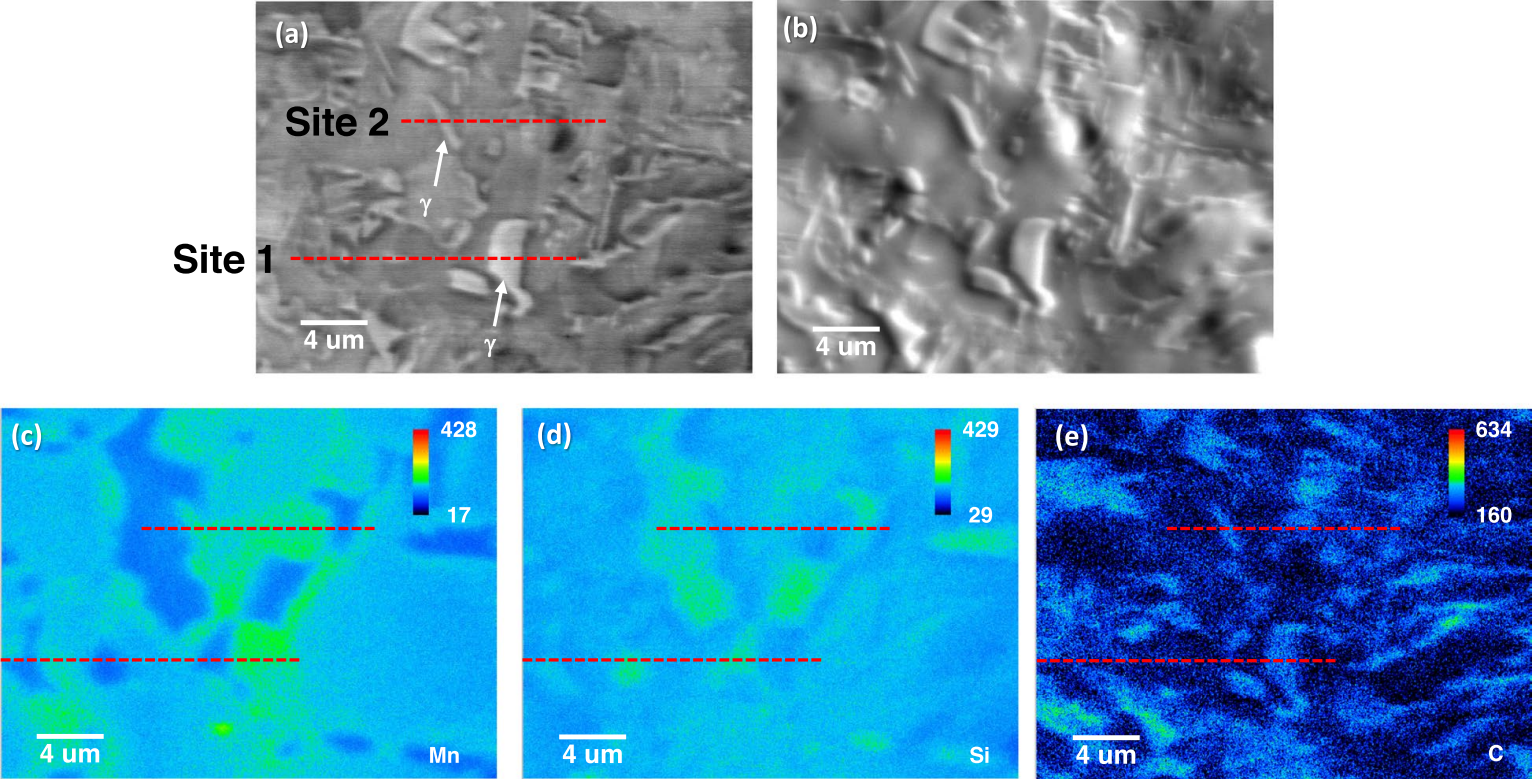
EPMA analysis of the retained γ; **a** backscattered electron and **b** SE images, and EPMA **c** Mn Kα, **d** Si Kα, and **e** C Kα maps. 20 kV, 3 × 10^− 7^ A, and 15 ms were used for the data acquisition

**Fig. 9:**
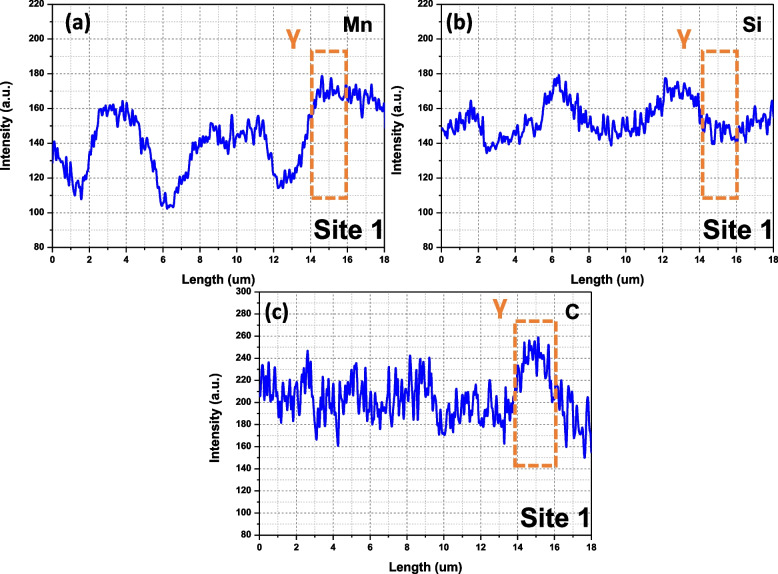
Extracted intensity profiles from site 1 in Fig. 7c to e

**Fig. 10:**
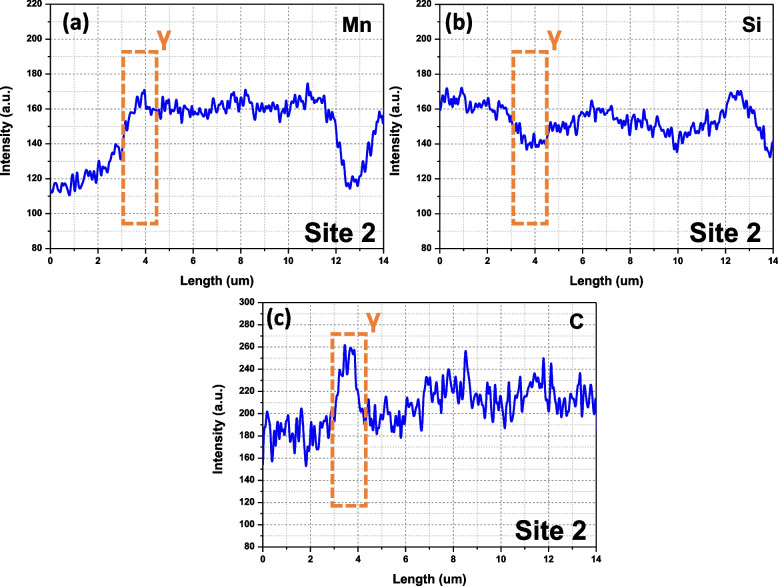
Extracted intensity profiles from site 2 in Fig. 7c to e

EPMA analysis was performed to measure the chemical composition of the retained γ. Figure 8a and b show backscattered electron and SE images, respectively. The corresponding EPMA maps are displayed in Fig. 8c to e. Two retained γ were selected, and the intensity profiles are extracted in Figs. 9 and 10. The X-ray intensities in the selected γ are listed in Table 2. The intensities are converted to the absolute composition using the obtained calibration curves in Fig. 5a to c. Interestingly, the thin γ film (site 2) shows higher C contents (1.39 wt.%) than that in blocky γ (1.29 wt.%).

**Table 2:** The X-ray intensity and the quantified compositions of the retained γ

Site	Intensity			Composition (wt.%)		
	Mn	Si	C	Mn	Si	C
**1**	**168.33**	**145.15**	**241.89**	**1.87**	**1.79**	**1.29**
**2**	**158.94**	**143.40**	**248.64**	**1.75**	**1.74**	**1.39**
**Average**	**163.64**	**145.28**	**245.27**	**1.81**	**1.77**	**1.34**

**Fig. 11:**
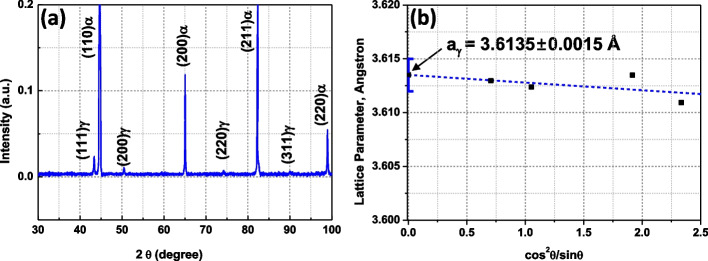
XRD spectrum and the lattice parameter-cos^2^θ/sinθ plot

The C contents are indirectly investigated by the lattice parameter of γ. The precise lattice parameter of γ was obtained from the XRD spectrum in Fig. 11a. Each lattice parameter obtained from specific planes of γ is plotted as a function of cos^2^θ/sinθ (Cullity and Stock 2001), and the precise lattice parameter (a_γ_) is obtained to 3.6135 ± 0.0015 Å (Fig. 11b). The lattice parameter of γ and the individual chemical composition show the following relationship (Seol et al. 2012);

9 $${\mathrm a}_{\mathrm\gamma}\left(\mathrm A\right)=3.5720\;+\;0.033{\mathrm W}_{\mathrm C}+0.0012{\mathrm W}_{\mathrm{Mn}}-0.00157{\mathrm W}_{\mathrm{Si}},$$

where W_i_ is the chemical composition (wt.%) of element i. Based on Eq. 9, the carbon contents in the retained γ were calculated. Mn and Si contents were used as the average values in Table 2. The calculated C content is 1.26 wt.%. Considering the difficulty of C quantification in energy dispersive spectroscopy, the EPMA quantification of the C content (average value) is relatively accurate, with a deviation of 0.08 wt% from the XRD result. The slightly higher C content in EPMA results probably originates from the different C contamination amounts among the observed specimens.

## Conclusion

The practical aspect of EPMA quantification was studied to evaluate the chemical composition of the retained γ in a Fe-1.5Mn-1.5Si-0.25C (wt.%) alloy. The followings are the conclusion obtained from this study;

1. The standard specimens show local inhomogeneity of chemical compositions. The EPMA calibration curves are successfully built by wide-range mapping and averaging the standard specimens.
2. Through studies on the various acquisition conditions, the following relationship among each condition was drawn;

$${\boldsymbol{I}}_{\boldsymbol{i}}^{\prime}\left({\textbf{p}}_{\textbf{2}},\,{\textbf{t}}_{\textbf{2}}\right)={\boldsymbol{I}}_{\boldsymbol{i}}\left({\textbf{p}}_{\textbf{1}},\,{\textbf{t}}_{\textbf{1}}\right)\times \left({\textbf{p}}_{\textbf{2}}/{\textbf{p}}_{\textbf{1}}\right)\times \left({\textbf{t}}_{\textbf{2}}/{\textbf{t}}_{\textbf{1}}\right).$$

- A calibration curve can be converted to a new calibration curve that corresponds to the new acquisition condition using the above relationship.

3. The chemical compositions of the retained γ were investigated in a Fe-1.5Mn-1.5Si-0.25C (wt.%) alloy using an EPMA. The obtained calibration curves were used for converting the X-ray intensity to the composition. The film type retained γ shows higher C content (1.39 wt.%) than the blocky type (1.29 wt.%).
4. The C content of the retained γ was calculated using the precise lattice parameter obtained from the XRD spectrum. The calculated C content (1.26 wt.%) shows good coherency with the C contents obtained from EPMA.

## Data Availability

The datasets used and/or analyzed during the current study are available from the corresponding author on reasonable request.
